# Determinants of disposal of child faeces in latrines in urban slums of Odisha, India: a cross-sectional study

**DOI:** 10.1093/trstmh/try142

**Published:** 2019-01-21

**Authors:** Fiona Majorin, Corey L Nagel, Belen Torondel, Parimita Routray, Manaswini Rout, Thomas F Clasen

**Affiliations:** 1 Department of Disease Control, Faculty of Infectious and Tropical Diseases, London School of Hygiene and Tropical Medicine, London, UK; 2 OHSU/PSU School of Public Health, Oregon Health and Science University, Portland, OR, USA; 3 Department of Environmental Health, Rollins School of Public Health, Emory University, Atlanta, GA, USA

**Keywords:** child faeces, cross-sectional study, India, sanitation, WASH

## Abstract

**Background:**

Even among households that have access to improved sanitation, children’s faeces often do not end up in a latrine, the international criterion for safe disposal of child faeces.

**Methods:**

We collected data on possible determinants of safe child faeces disposal in a cross-sectional study of 851 children <5 y of age from 694 households in 42 slums in two cities in Odisha, India. Caregivers were asked about defecation and faeces disposal practices for all the children <5 y of age in the household.

**Results:**

Only a quarter (25.5%) of the 851 children’s faeces were reported to be disposed of in a latrine. Even fewer (22.3%) of the 694 households reported that the faeces of all children <5 y of age in the home ended up in the latrine the last time the child defecated. In multivariate analysis, factors associated with being a safe disposal household were education and religion of the primary caregiver, number of children <5 y of age in the household, wealth, type and location of the latrine used by the household, household members >5 y of age using the latrine for defecation and mobility of children <5 y of age in the household.

**Conclusions:**

Few households reported disposing of all of their children’s faeces in a latrine. Improving latrine access and specific behaviour change interventions may improve this practice.

## Introduction

Poor sanitation is a major cause of faecal–oral diseases, including diarrhoea, which is responsible for >1.6 million deaths annually.^1^ In 2015, 2.3 billion people did not have access to at least basic sanitation worldwide, including 892 million people that practiced open defecation.^2^ In India, 40% of its population practiced open defecation and only 44% used at least basic facilities.^2^

Child faeces represents a particular threat to human health, as young children have the highest incidence of enteric infections^3^ and their faeces are most likely to contain transmissible pathogens.^4^ In addition, children tend to defecate in places where other children, who are particularly vulnerable due to their immature immune systems and exploratory behaviours,^5^ could be exposed.^6^ A review found that child faeces disposal behaviours that are considered risky were associated with a 23% increase in the risk of diarrhoeal diseases (relative risk [RR] 1.23 [95% confidence interval {CI} 1.15 to 1.32]).^7^ A recent study analysing Demographic and Health Survey (DHS) data from 34 countries found that child faeces disposal practices were strongly associated with child growth. The study found that improved child faeces disposal practices (child faeces disposed into improved latrines) were associated with reduced levels of child stunting and underweight and increases in height-for-age Z scores and weight-for-age Z scores.^8^

Our research suggests that there are multiple sources of exposure from child faeces beyond defecation and disposal.^9^ These include unhygienic collection of faeces or cleaning of surfaces when children defecate on the floor or ground (diapers and potties being rare in many low-income settings) and inadequate hand-washing after disposing of the faeces. However, international monitoring currently defines ‘safe disposal’ of child faeces solely on the basis of whether the faeces ends up in a latrine, either because the child defecated in a latrine or the faeces were subsequently deposited there.

Even in settings with improved sanitation or ‘basic sanitation’,^2^ child faeces are often not disposed of in latrines.^10–15^ This creates a potentially important source of exposure to faecal pathogens. A report by the World Bank Water and Sanitation Programme (WSP), presenting analysis from the latest available Multiple Indicator Cluster Surveys (MICS) and DHSs (2006–2012) found that in 15 of 26 locations >50% of households reported unsafely disposing of faeces from children <3 y old (not into a latrine); the percentage of children whose faeces ended up in improved sanitation facilities was even lower.^12^ In India, the latest available DHS (2015–2016) found that the faeces of only 34.7% of children end up in a latrine (22.0% from the child defecating directly in the latrine and 12.7% from subsequent disposal in the latrine); an additional 1.5% were buried, until recently also considered ‘safe disposal’.^13^ A previous cross-sectional study of child faeces disposal practices among rural households in villages in the State of Odisha, where the Total Sanitation Campaign (TSC) had been implemented at least 3 y before, found that 81.4% of child faeces were disposed of unsafely, with the majority of faeces reported being deposited with solid waste.^11^ However, that study did not address the context in urban slums, which were not covered by the TSC and which are likely to present additional challenges due to the absence of land and space for building latrines, higher reliance on more distant shared and public facilities and greater population density and migration that can impact social norms. The health risks presented by children’s faeces are likely to be greater in urban slums due to increased opportunities for exposure and disease transmission.^16^

While the government of India has endeavoured to improve sanitation through a series of initiatives aimed at reducing open defecation, evaluations of these have found limited impacts on child faeces disposal practices. In one such evaluation, the intervention increased the safe disposal of child faeces from 1.1% at baseline to 10.4% in intervention households, compared with 3.1% in the control households (RR 3.34 [95% CI 1.99 to 5.59]).^10^ In another study, the intervention also resulted in an increase in safe child faeces disposal of 9 percentage points (27% intervention vs. 18% control; p<0.001).^17^ Notably, the sanitation programs evaluated were aimed chiefly at increasing latrine coverage; they included few behaviour change initiatives to increase latrine use, including use by children or for safe disposal of child faeces. While these studies showed some improvements in child faeces disposal, the majority of faeces still ended up in the environment.

Investigating factors that are associated with child faeces disposal may help in understanding the reasons for the low prevalence and identify potential ways to improve these behaviours. Factors that have previously been found to be associated with disposal of child faeces into a latrine include child characteristics and practices (mobility category, defecation site of the child, child age), factors related to water and sanitation access and use (number of years of latrine ownership, access to a latrine in the compound, type of latrine, consistency of adult latrine use, presence/ownership of child faeces management tools, presence of a hand-washing facility and type of water source) and socio-economic and demographic characteristics (urban residence, household wealth, household head’s education, number of children <5 y of age in the household, mother’s education, caregiver’s/mother’s age, attendance at health education sessions, media exposure, religion, caste/tribe of head of household).^10–15,18–23^

Informal settlements in urban settings present particular sanitation challenges.^5,24^ We undertook this study to examine the factors associated with the disposal of child faeces in latrines (‘safe disposal’) in urban slums in Orissa, India.

## Materials and methods

### Study design and setting

Details of the study design and setting have been described elsewhere.^9^ Briefly, the study followed a cross-sectional design. The data collection took place in July and August 2014. Households were selected using an adaptation of the Extended Program of Immunization (EPI) sampling method.^25^ Households eligible for inclusion in the study were required to meet the following eligibility criteria: have at least one child <5 y of age with a primary caregiver >18 y of age and the primary caregiver reported having access to sanitation facilities (individual household latrines, shared or communal facilities) or belonged to a slum with communal sanitation facilities (even if the respondent reported no one in the household used these). Households that otherwise met such eligibility criteria were nevertheless excluded from the study if the primary caregiver was an Accredited Social Health Activist, an *anganwadi* (government sponsored child-care and mother-care centre) worker or a person who had worked for health promotion campaigns. The number of participating households in each slum varied due to the varying sizes of the slums and the availability of households with children <5 y of age at the time of the visit. Respondents were the primary caregivers (defined as ‘the one who usually cares for the child’) of the youngest child <5 y of age in each household. Households that were locked, where the primary caregiver was unavailable at the time of the visit, that did not meet the eligibility criteria or that refused to participate were not enrolled and the researchers would go to the next household on the left until they found one that met the eligibility criteria.

### Slum selection

The informal settlements (slums) were selected from a list of 23 slums in Cuttack and 39 slums in Bhubaneswar.^26^ The selection criteria for the slums was that they had at least 33 households with access to either individual household latrines or functioning community latrines.^26,27^ We excluded three leprosy colonies from our list of eligible slums as well as slums in which pilot activities were previously conducted. This selection process resulted in 20 eligible slums in Cuttack and 28 eligible slums in Bhubaneswar.

### Sample size calculation

The primary outcome for this cross-sectional study is the proportion of children <5 y of age whose faeces are disposed of safely (defined here as defecation or disposal in a latrine). Based on previous studies,^10,11,28,29^ the sample size was calculated using the average of 30% safe disposal. Using simple random sampling, the average of 30% safe disposal of child faeces led to a sample size for frequency in a population of 323 households (assuming one child per household) (with 95% confidence).^30^ The sample size calculation was adjusted to account for clustering, with an intracluster correlation coefficient of 0.06 based on previous work in rural Odisha.^31^ Based on the different sample size calculations in different scenarios, 20 households in 35 clusters (a total of 700 households) was chosen to be the best logistical option. The study was not separately powered for each city but for 35 slums in total. As it was not always possible to find 20 eligible households in each selected slum, we continued selecting slums in the order in which they had been randomly ordered until we reached our target sample size of 700 households. This resulted in the data being collected in 42 slums: 22 in Bhubaneswar and 20 in Cuttack.

### Data collection tools

Data collection tools included a structured survey and checklist for spot checks. The survey included questions on socio-economic and demographic factors, access to sanitation, water and hygiene facilities, availability of potties and diapers and exposure to messages about child sanitation or hygiene. Questions about defecation place and faeces disposal method for the last time each child <5 y of age defecated^11^ were included, using wording as per the core questions of the World Health Organization/United Nations Children’s Fund Joint Monitoring Programme on Water and Sanitation (JMP).^32^ The age and mobility (whether the child can or cannot walk) of the children, whether they were exclusively breastfed and the consistency of their faeces (solid, liquid, semisolid) the last time they defecated were also recorded. The questions on defecation and disposal practices for the last time the children defecated were asked for all the children <5 y of age in each household (defined as sharing the same cooking pot). Data were also collected on the age and usual defecation places of each family member >5 y of age.^33^

Spot-checks were done to determine the type of latrine (flush/pour flush with pit/closed sewer system, flush/pour flush without pit/open sewer system, pit latrine with slab or other) reported by the households as the one used the majority of the time, to check the presence of a potty in the household, whether children were wearing a diaper and to check the availability of soap and water at the specific place identified by participants that was used for hand washing after disposal of child faeces.

The survey, information sheet and consent forms were written in English and then translated into Odia, the local language. A researcher bilingual in Odia and English evaluated the translation. All the researchers who conducted the surveys were fluent Odia speakers.

### Data entry and analysis

Data were double entered using EpiData 3.1 (EpiData Association, Odense, Denmark) and analysed using STATA version 14 (StataCorp, College Station, TX, USA). Child faeces disposal was categorized as safe if children’s faeces were reported to have ended up in a latrine, either by the child defecating directly into the latrine or by subsequent disposal in a latrine. Consistent with JMP definitions for safe disposal, the latrine could be either improved or unimproved.^32^ The analysis was performed at the household level, whether a household practiced safe disposal of all the children’s faeces (‘safe disposal household’) or none or only a portion of the children’s faeces were disposed of in a latrine (‘unsafe disposal household’).

An asset index was created by combining household information on the number of rooms for sleeping, household construction type and ownership of items (watch/clock, pressure cooker, radio, television, dish antenna, refrigerator, mobile phone, mattress, bed/cot, chair, table, sewing machine, bicycle, motorbike) using polychoric principal component analysis.^34^ The wealth score was divided into tertiles. The number of room for sleeping was missing two values and these were replaced by the average value for households with the same number of total rooms. The type of latrine (improved or unimproved) and location of the latrine were combined into a variable with three levels: unimproved outside compound, unimproved inside compound or in/attached to the dwelling and improved latrine (of which seven were outside the compound).

Bivariate analyses were conducted to assess the association of safe disposal households with each of the possible covariates collected. Polychoric correlations were used to check correlations between all variables and collinearity diagnostics were checked. All variables with a p-value <0.25 (Wald) in the bivariate analysis were considered for inclusion in the multivariate analysis. Variables that were not significant (p<0.1) in the full model were removed one at a time; checking the odds ratios (ORs) in the model did not change >20%. This was conducted until all insignificant variables were excluded from the model. Variables initially excluded after the bivariate analysis were then checked for significance and included if p<0.1. Finally, interactions were investigated between wealth and latrine type.^35^ Generalized estimating equations with robust standard errors were used to calculate ORs and accounted for clustering at the slum level using an exchangeable correlation matrix.

## Results

### Study population and child faeces disposal practices

A total of 694 households with 852 children <5 y of age were enrolled from 42 slums. There was an average of 16.5 respondents per slum (range 3–20). Most households (554/694 [79.8%]) had just one child <5 y of age, while 140 households had more than one child <5 y of age; 18.0% of households had two children <5 y of age and 2.1% had more than two children <5 y of age. Complete data on defecation behaviours were available for 851 children; the missing child belonged to a household with three children and is considered for this analysis as a household with two children.

Overall, 25.5% (95% CI 22.7 to 28.5) of the 851 children were reported to have their faeces end up in the latrine the last time they defecated (faeces of 217 children from 200 households). Most of these (20.3% [95% CI 17.8 to 23.2]) defecated directly into the latrine while the others had faeces deposited there after defecating elsewhere. Notably, only 13.5% (95% CI 11.4 to 16.0) of children had faeces that ended up in improved latrines (improved disposal).

At the household level, 22.3% of households disposed of all the faeces of children <5 y of age in the latrine the last time the child defecated (155/694; 142 households had 1 child, 13 households had 2), 6.5% (45/694) of households disposed of some of the children’s faeces in the latrine (38 households disposed of 50% of the children’s faeces in the latrine, 4 households disposed of 66.7% and 3 households disposed of 33.3%), 71.2% (494/694) disposed of none of the children’s faeces in the latrine (412 with 1 child, 75 with 2 children, 4 with 3 children, 3 with 4 children).

### Bivariate analysis

**Figure 1.**
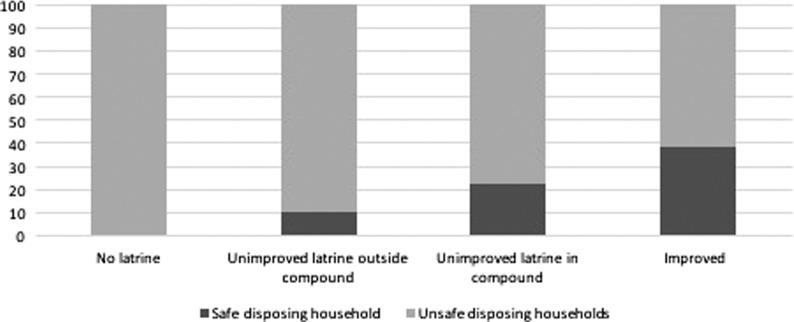
Bar chart proportions of safe disposing households by types of sanitation facilities.

In the bivariate analysis the following factors were found to be associated with safe disposal households (Wald p<0.25): education, age, religion and occupation of the primary caregiver, number of children <5 y of age in the household, wealth, location of the drinking water source, type and location of the latrine, having heard or seen a message about child sanitation or hygiene, use of the latrine by household members >5 y of age and mobility of the children in the household (Table 1). Of those factors, all but age and religion of the primary caregiver were significant at the 0.05 level. Certain other variables were also associated with safe disposal (attending nursery [*anganwadi*], breastfeeding and age), but these were excluded due to their collinearity with mobility (Supplementary Table 1). Having a place to wash hands with soap and water was excluded since the question was only asked to caregivers who disposed of their child’s faeces (i.e. the child did not defecate directly in the latrine and faeces were not left in the open); what is used to wash a child’s bottom was also excluded because of a lack of reported data. Whether the defecation place of children <5 y of age was improved or not was also associated with the outcome. However, this was not included in the multivariate analysis because it excluded the 114 households in which all children used the latrine. None of the households that reported not using sanitation facilities, despite having access to communal facilities, practiced safe child faeces disposal (see Figure 1). Thus 55 children from 45 households were excluded from the multivariate analysis, resulting in a sample of 796 children in 649 households.

**Table 1.** Bivariate analysis assessing association between risk factors and safe disposal households

Variables	Safe disposal households						
	N	Total	%	OR	Lower CI	Upper CI	p-Value (Wald)
Education of primary caregiver	694						
Illiterate/no formal schooling	14	112	12.5	Ref			
Some/completed primary school	13	135	9.6	0.66	0.34	1.30	0.229
Completed secondary school	86	350	24.6	1.63	0.95	2.80	0.078
Any level of higher education	42	97	43.3	3.59	1.95	6.60	<0.001
Age of primary caregiver (y)	694						
18–24	48	264	18.2	Ref			
25–29	57	257	22.2	1.24	0.81	1.89	NS^a^
≥30	50	173	28.9	1.77	1.11	2.84	0.017
Religion of primary caregiver	694						
Hindu	140	654	21.4	Ref			
Muslim/Christian^b^	15	40	37.5	2.30	0.84	6.25	0.104
Caregiver has a job	694						
No	139	632	22.0	ref			
Yes^c^	16	62	25.8	1.58	1.04	2.40	0.032
Number of children <5 y of age in the household	694						
1	142	554	25.6	Ref			
2–4	13	140	9.3	0.35	0.22	0.58	<0.001
Number of people >5 y of age living in the household	694						
1–2	39	165	23.6	Ref			
3–4	53	253	21.0	0.89	0.56	1.42	NS^a^
5–6	32	157	20.4	0.93	0.64	1.35	NS^a^
7–16	31	119	26.1	1.23	0.72	2.09	NS^a^
Wealth	694						
Poorest	39	232	16.8	Ref			
Middle	43	231	18.6	0.98	0.59	1.63	NS^a^
Least poor	73	231	31.6	1.90	1.17	3.10	0.009
Gender of head of household	694						
Female	22	127	17.3	Ref			
Male	133	567	23.5	1.28	0.83	1.99	NS^a^
Ownership of residence	694						
Owner	110	506	21.7	Ref			
Tenant	45	188	23.9	1.02	0.73	1.42	NS^a^
Time in house (y)	692						
<1	11	43	25.6	Ref			
1–5	30	115	26.1	1.00	0.45	2.24	NS^a^
≥5	114	534	21.4	0.94	0.50	1.76	NS^a^
Location of drinking water (98.8% improved)	693						
Outside compound	49	337	14.5	Ref			
In compound	37	135	27.4	1.83	1.09	3.09	0.023
In dwelling	69	221	31.2	2.34	1.45	3.77	<0.001
Type of latrine^d^	649						
Unimproved latrine outside compound	26	248	10.5	Ref			
Unimproved latrine in compound	36	160	22.5	2.21	1.23	3.96	0.008
Improved	93	241	38.6	4.73	2.77	8.10	<0.001
Ownership of a potty	694						
No/unable to show^e^	141	648	21.8	Ref			
Yes observed	14	46	30.4	1.34	0.69	2.59	NS^a^
Buy diapers sometimes	694						
No/don’t know	79	366	21.6	Ref			
Yes	76	328	23.2	0.89	0.57	1.39	NS^a^
Hand-washing place^f^	529						
No specific place	8	159	5.0	Ref			
Hand-washing facility	2	140	1.4	0.25	0.037	1.68	0.154
Hand-washing facility with soap and water	33	230	14.4	2.62	1.29	5.33	0.008
Wash child’s bottom^g^	681						
Use water	102	489	20.9	Ref			
Use water and soap	44	125	35.2	2.01	1.38	2.92	<0.001
Use cloth/wipe/paper	4	67	6.0	0.19	0.06	0.59	0.004
In the last 6 months, have heard/seen any messages about child sanitation or hygiene^h^	694						
No	95	477	19.9	Ref			
Yes	60	217	27.7	1.38	1.01	1.91	0.046
Ever heard of a program promoting the use of latrines by children?	694						
No/don’t know	143	642	22.3	Ref			
Yes	12	52	23.1	0.92	0.39	2.18	NS^a^
Summary variables per household for persons >5 y of age							
All members of household >5 y of age use latrine always	649						
No	5	116	4.3	Ref			
Yes	150	533	28.1	5.84	1.81	18.83	0.003
Summary variables per household for persons <5 y of age							
Proportion of males and females per household <5 y of age	694						
Female≥male	86	385	22.3	Ref			
Female<male	69	309	22.3	1.03	0.74	1.43	NS^a^
Proportion of mobility category in household	694						
All/some pre-ambulatory	11	211	5.2	ref			
All ambulatory	144	483	29.8	7.07	3.55	14.08	<0.001
Defecation site of persons <5 y of age	580						
All/some unimproved^i^	24	384	6.3	Ref			
All semi-improved^j^	7	137	5.1	0.67	0.25	1.78	NS^a^
All improved^k^	8	34	23.5	3.93	1.60	9.62	0.003
Mixed semi-improved/improved/use latrine	2	25	8.0	1.31	0.33	5.24	0.701
Proportion of solid feces^l^	462						
All liquid	1	44	2.3	Ref			
All solid	31	333	9.3	5.22	0.57	47.76	0.144
All semisolid	3	64	4.7	2.89	0.23	36.26	0.41
Some liquid/solid/semisolid	0	19	0	Dropped			
All didn’t know/didn’t see	0	2	0	Dropped			

Ref: reference.

^a^p-value>0.25.

^b^32 Muslims, 8 Christians.

^c^Mostly day labour (44/62), private job (10/62), government job (4/62), business (4/62).

^d^Excludes 45 households who practice open defecation, none are safe disposal households. Outside compound includes in neighbour’s compound or dwelling, inside compound includes attached/in dwelling, improved latrines include seven that were out of the compound.

^e^Seven households did not show the potty, 379 respondents had never heard of a potty.

^f^Only for those who responded or demonstrated disposing of faeces of at least one child in household (i.e. child did not defecate in latrine or faeces were not left in the open); 4 missing, 2 reported no hand washing.

^g^Only for those who wash (3 said the child washed himself and 10 said they did not wash). Water includes water and powder; soap includes Dettol; cloth includes cloth with Dettol or water or coconut oil.

^h^From television, radio, poster/wall painting, newspaper/magazine or other.

^i^On ground or floor in latrine cubicle, roadside, riverside, field, side path, in compound, household, drain, bathroom.

^j^On paper, polythene, cloth, oil cloth or plank.

^k^In potty, nappy, pants or diaper.

^l^Restricted to households in which none of the children defecated in the latrine or drain or were left in the open. Thus the safe disposal households only include those where the faeces of all the children in the household were deposited in the latrine when the child defecated elsewhere (n=35).

### Multivariate analysis

**Figure 2.**
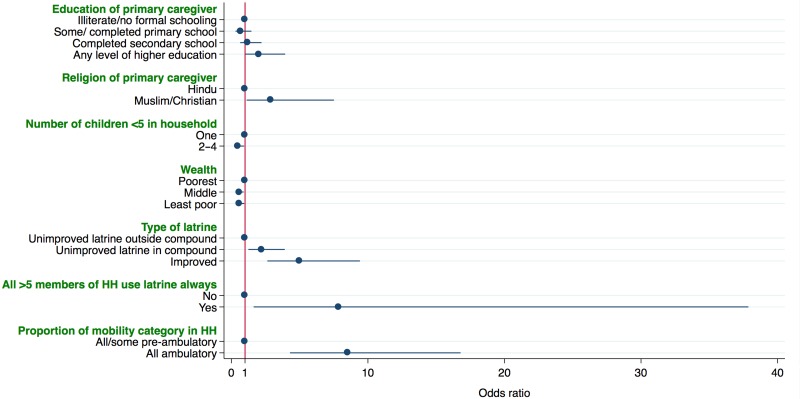
Odds of being a safe disposal household.

The multivariate analysis resulted in the following variables being significantly associated with being a safe disposal household: education and religion of the primary caregiver, number of children <5 y of age in the household, wealth, type and location of the latrine, defecation behaviours of the household members >5 y of age and the mobility of children in the house (Table 2, Figure 2). A caregiver with higher education than secondary school was associated with increased odds of being a safe disposal household compared with caregivers who were illiterate or had no formal schooling (adjusted OR [aOR] 2.01 [95% CI 1.03 to 3.94]). Being Muslim or Christian increased the odds of being a safe disposal household (aOR 2.89 [95% CI 1.11 to 7.51]). Having more than one child decreased the odds of being a safe disposal household (aOR 0.46 [95% CI 0.23 to 0.93]). Being a middle or least poor household decreased the odds of being a safe disposal household compared with the poorest households (middle aOR 0.54 [95% CI 0.33 to 0.89; least poor aOR 0.55 [95% CI 0.32 to 0.94]). Households using an unimproved latrine located in the compound or in/attached to the dwelling (aOR 2.20 [95% CI 1.24 to 3.91]) and using an improved latrine increased the odds of being a safe disposal household (aOR 4.98 [95% CI 2.63 to 9.42]) compared with households using unimproved latrines outside the compound. Households where all the members >5 y of age were reported to use the latrine always had higher odds of being a safe disposal household (aOR 7.84 [95% CI 1.63 to 37.86]). Households where all the children <5 y of age were ambulatory had 8.49 times the odds of being a safe disposal household (aOR 8.49 [95% CI 4.29 to 16.79]).

**Table 2.** Adjusted associations between risk factors and safe disposal households (n=649)

Variables	aOR	Lower CI	Upper CI	p-Value (Wald)
Education of primary caregiver				
Illiterate/no formal schooling	Ref			
Some/completed primary school	0.66	0.30	1.47	0.311
Completed secondary school	1.19	0.64	2.21	0.577
Any level of higher education	2.01	1.03	3.94	0.042
Religion of primary caregiver				
Hindu	Ref			
Muslim/Christian	2.89	1.11	7.51	0.029
Number of children <5 y of age in the household				
1	Ref			
2–4	0.46	0.23	0.93	0.031
Wealth				
Poorest	Ref			
Middle	0.54	0.33	0.89	0.017
Least poor	0.55	0.32	0.94	0.029
Type of latrine				
Unimproved latrine outside compound	Ref			
Unimproved latrine in compound	2.20	1.24	3.91	0.007
Improved	4.98	2.63	9.42	<0.001
All members of the household >5 y of age use latrine always				
No	Ref			
Yes	7.84	1.63	37.86	0.01
Proportion of mobility category in household				
All/some pre-ambulatory	Ref			
All ambulatory	8.49	4.29	16.79	<0.001

## Discussion

The factors found to be associated with being a safe disposal household are similar to those of previous studies. Azage and Haile^18^ found that an increase in caregiver education and a lower number of children in the household were associated with safer disposal. The consistency of adult toilet use has also been found to be associated with safe disposal in other recent studies.^14,22^

Being a Christian or Muslim was associated with higher odds of safe disposal. This was also found in a recent study analysing the latest India DHS data, which found that Muslim households and ‘other religion’ households had lower odds of unsafe disposal than Hindu households.^19^ This finding may be explained by Hindu religious rituals that may prevent safe disposal in a latrine, such as cleaning of clothes after entering the latrine.^36^ The Sanitation Quality, Use, Access and Trends survey also found that religion was associated with use of the latrine, with Muslims using their latrine more than Hindus.^37,38^

In this study we found that being from a wealthier household was associated with poorer child faeces disposal practices, which is contrary to other studies.^12,14,15,18,19^ This may be due to confounding of the relationship between wealth and the outcome or that the assets used to generate the wealth categories do not represent wealth accurately.

The strong association of being a safe disposal household with using an improved latrine has been found in other studies.^12,18,19,21,23^ Additionally, in this study we subgrouped unimproved latrines by distance and found that unimproved latrine users were more likely to be a safe disposal household if the latrine they used was nearer to their dwelling, which may be due to the convenience of disposing of faeces or training children to use a latrine if it’s closer to the house. We have previously described that the reported age of latrine training was younger for children in households using private and shared latrines compared with communal latrines.^9^ In addition, for communal latrine user households, it may not be seen as adequate or practical for children to use them.^5^ A recent study in Accra, Ghana found that children were unlikely to use public toilets.^39^ A further study in Accra also found that disposal of faeces of children <5 y of age was more common in households with a within-compound latrine than in households that relied on public latrines.^40^

The mobility of children is strongly associated to safe disposal. This is likely due to the fact that most safe disposal is due to ambulatory children directly defecating in the latrine. This has also been found in previous studies in rural Odisha.^10,11^ Similarly, an increase in safe disposal with increasing age of the children has been found in other studies.^12,14,18–22^ This suggests that there is a need to design interventions for younger children who are defecating elsewhere than the latrine.

### Limitations

While we have used the definition of safe disposal promoted by international monitoring (i.e. disposal of child faeces in any latrine, improved or unimproved), we would not recommend this classification of safe disposal. Children’s faeces should at least be considered to be as risky as those of adults and thus should be treated in the same way with regards to disposal.

This article only focuses on associations between households that dispose of all of the children’s faeces in a latrine and possible determinants. However, child faeces management contains several critical points beyond the final disposal place that need to be mitigated to avoid exposure, including the place of defecation, cleaning of that place and hygiene behaviours.^9^ Furthermore, the study quantified safe disposal using questions about the last time each child defecated, but this behaviour is likely to change and has not been found to be consistent in other studies.^10,22^

The results of this study are only generalizable to the population included in the study. Also, this study was conducted during the rainy season and thus behaviours may differ from other seasons. In addition, it has been found that participants overreport ‘desirable’ behaviours of child faeces disposal when data are collected using questionnaires compared with structured observations.^41,42^ We tried to minimize this by using questions about the last time children defecated.^32^ In addition, recent evidence suggests that reported and observed behaviour were very similar.^43^

## Conclusions

Few households reported disposing of all of their children’s faeces in a latrine. Various characteristics of study participants and their households were associated with the safe disposal of child faeces. Many of these, such as education and religion of the primary caregiver, household wealth, number and ambulatory status of children <5 y of age in the household, are either not amenable to or cannot be changed by short-term interventions. Others, however, such as access, type and proximity to latrines and whether other household members use latrines, are within the purview of sanitation programs. Such programs, however, must address not only deficiencies in latrine coverage, but also deficiencies in practices. Further research should also investigate whether these behaviour change interventions could be enhanced by provision devices that can facilitate safe disposal (e.g. nappies, scoops, potties) while also minimizing other sources of exposure.

## Supplementary Material

Supplementary Data
