# Supplementary material for: Determinants of disposal of child faeces in latrines in urban slums of Odisha, India: a cross-sectional study
Source: Trans R Soc Trop Med Hyg. 2019 Jan 21;113(5):263–72. doi: 10.1093/trstmh/try142 (PMC6515899; doi:10.1093/trstmh/try142)
Supplement: Supplementary Data [file try142_determinants_of_cfd_in_latrines_sup_table_1.docx]

**Supplementary table 1: Additional variables from the bivariate analysis, assessing association between risk factors and safe disposal households**

|  | **Safe disposing household** | | | | | | |
| --- | --- | --- | --- | --- | --- | --- | --- |
| **Variables** | **N** | **Total** | **%** | **OR** | **lower CI** | **upper CI** | **P-value (Wald)** |
| **Age composition** | **694** |  |  |  |  |  |  |
| Some/ all are infants (<1 yo) | 9 | 151 | 6.0 | ref |  |  |  |
| None are infants | 146 | 543 | 26.9 | 5.90 | 2.80 | 12.44 | <0.001 |
| **Proportion of <5 who go to *anganwadi*** | **694** |  |  |  |  |  |  |
| All/some never attend | 77 | 513 | 15.0 | ref |  |  |  |
| All attend (always/sometimes) | 78 | 177 | 44.1 | 4.59 | 3.01 | 6.98 | <0.001 |
| Missing | 0 | 4 | 0 | _ |  |  |  |
| **Proportion exclusively breastfed** | **694** |  |  |  |  |  |  |
| All/some exclusively breastfed | 2 | 58 | 3.5 | ref |  |  |  |
| Some mixed and some not breastfed | 8 | 93 | 8.6 | 2.99 | 0.75 | 11.97 | 0.122 |
| All mixed food (breastfeeding and other) | 47 | 311 | 15.1 | 4.54 | 1.14 | 18.08 | 0.032 |
| All other than breastfed | 98 | 231 | 42.4 | 18.22 | 4.58 | 72.53 | <0.001 |
| Missing | 0 | 1 | 0 | _ |  |  |  |
